# Recent Insights into Bioactive Dichalcogen Derivatives: From Small Molecules to Complex Materials

**DOI:** 10.3390/ijms26062436

**Published:** 2025-03-08

**Authors:** Leire Gaviria-Soteras, Arun K. Sharma, Carmen Sanmartín, Daniel Plano

**Affiliations:** 1 Department of Pharmaceutical Sciences, University of Navarra, Irunlarrea 1, E-31008 Pamplona, Spain; lgaviria@alumni.unav.es (L.G.-S.); dplano@unav.es (D.P.); 2 Department of Molecular and Precision Medicine, Penn State Cancer Institute, CH72, 500 University Drive, Hershey, PA 17033, USA; asharma1@pennstatehealth.psu.edu; 3 Instituto de Investigación Sanitaria de Navarra (IdiSNA), Irunlarrea 3, E-31008 Pamplona, Spain

**Keywords:** diselenide, ditelluride, therapeutic activity, small molecules, nanocarriers

## Abstract

Organodichalcogenides have been explored due to their therapeutic properties. They have been demonstrated to be active against several diseases such as cancer, bacteria, viruses, parasites, or neurological diseases. Among the different classes of dichalcogenides, disulfide derivatives have been widely studied, and many studies cover their therapeutical use. For this reason, this review includes the latest studies of diselenides and ditellurides derivatives with biological applications. With this aim, several bioactive small molecules containing the diselenide or ditelluride bond in their structure have been discussed. Furthermore, it should be highlighted that, in recent years, there has been an increasing interest in the development of nanomaterials for drug delivery due to their therapeutic advantages. In this context, diselenide and ditelluride-containing nanocarriers have emerged as novel approaches. The information compiled in this review includes small molecules and more complex materials containing diselenide or ditelluride bonds in their structure for different therapeutical applications, which could be helpful for the further development of novel drugs for the treatment of different diseases.

## 1. Introduction

Chalcogens comprise the elements of Group 16 of the periodic table: oxygen (O), sulfur (S) selenium (Se), tellurium (Te), and polonium (Po). Chalcogen atoms frequently act as nucleophiles, but some of them can also behave as electrophiles depending on their electronic features [1]. It has been shown that chalcogen bonds are involved in multiple protein–ligand interactions [2].

### 1.1. Chalcogens and Bioactivity

Living organisms have developed several strategies to incorporate chalcogen atoms, specifically S and Se, into bioactive natural products. Chalcogen-containing compounds have important biological functions and many of them are present in several drugs used to treat various diseases such as cancer or infections [3]. S is one of the most abundant elements on earth and it is present in all kingdoms of life. It is incorporated into proteins, vitamins, essential metabolites, and cofactors. Consequently, a plethora of research has been dedicated towards S-containing compounds for the treatment of several diseases, as it plays a crucial role in medicine [4,5,6,7,8,9,10].

On the other hand, the consecutive chalcogen Se, despite being much less abundant, is required for some essential functions [11]. Se is an essential microelement necessary for a wide variety of biological functions. Se, and more specifically the amino acid selenocysteine, is found in at least 25 human selenoproteins, which play a crucial role in regulating various biological functions, including the homeostasis of reactive oxygen species (ROS) and the biosynthesis of hormones [12]. It is very important to maintain adequate Se levels, since a deficiency of Se is associated with a wide variety of diseases and an excess of Se can present toxic effects [13]. In many aspects, S and Se have similar physical and chemical properties, as they share the same group in the periodic table and oxidation states [14]. However, the larger size of Se makes it more polarizable; therefore, Se bonds have lower bonding energy, resulting in faster bond-breaking reactions [11]. The greatest difference between the chemistry of both elements (S and Se) occurs in the redox reactions. Se is able to react with ROS in a readily reversible manner. Furthermore, the higher stability of selanyl radicals compared with thiyl radicals makes Se-containing proteins more able to withstand one-electron oxidation events [15]. Therefore, in recent years, Se is starting to replace S, which is reflected in an increase in the number of reported studies [16,17,18].

Regarding Te, it is the heaviest non-radioactive member within the chalcogen family. Traditionally, the major use of Te has been in metallurgy as an alloying agent with steel or cooper. However, in the last century, given its unique properties, Te compounds have been applied to other fields such as biomedicine [19]. Unlike the two chalcogens mentioned previously, there are very few examples where Te occurs in living organisms, which could be associated with its toxic effects. These toxic effects depend on the chemical form of Te. The biological chemistry of Te is just emerging [20]. However, during the last couple of decades, some interesting studies have been reported about its application as an element in drug development [21,22,23].

### 1.2. Diorganochalcogenides and Bioactivity

Dichalcogenide derivatives refers to molecules containing two covalently bonded chalcogens, that is to say, disulfides, diselenides, or ditellurides. These types of chemical structures represent a group of interest in medicinal chemistry.

A wide variety of natural occurring products that contain disulfide bonds have displayed potent biological activities, including anticancer [24,25,26,27], antibacterial [28,29,30], or antifungal activities [31]. Furthermore, the working mechanisms have also been elucidated, providing evidence of the key role of the disulfide group. This field has already been highly explored and there is a great variety of studies in relation to disulfide derivatives as bioactive agents [32].

On the other hand, organodiselenide derivatives have emerged as promising compounds due to their pharmacological activities such as anticancer [33], antibacterial [34,35], antifungal [36], and antiparasitic [37] activities, or for the treatment of neurodegenerative diseases [38]. Furthermore, they are among the most evaluated organoselenium compounds. Despite the similarity between S-S and Se-Se bonds, their chemical differences result in Se-Se-containing molecules having particular characteristics. The diameter of Se is 115 ppm, and its electronegativity is 2.55, while the diameter of S is 100 pm with an electronegativity of 2.58. As a consequence, the larger diameter of Se results in a lower bond energy (Se-Se = 172 kJ/mol) than the disulfide bond (S-S = 268 kJ/mol). Additionally, the redox potential of the Se-Se bond is lower than the S-S bond, making it more prone to reduction. Specifically, the Se/H_2_Se redox couple has a potential of −0.115 V, while the S/H_2_S redox couple is 0.144 V. Consequently, these differences in bond strength, and redox potential, among others, make the diselenide bond more redox-active than the disulfide bond, which has significant relevance in biological reactions [39].

Regarding the mechanisms of action of diselenides, there are many of them, which, unfortunately, are multidirectional and not fully understood. However, their actions mainly arise from the high redox activity of the produced metabolites. Se-Se is a redox-responsive chemical bond. Cancer cells generate higher levels of ROS and glutathione (GSH) compared to normal cells, which provide redox characteristics of their microenvironment. In this redox microenvironment, diselenide bonds undergo oxidation by ROS to form selenic and seleninic acid (-SeOOH), which are more reactive than its S analogues. On the other hand, the reduction by GSH to yield selenol (-SeH) for diselenides is faster than the S-S bond due to the weaker Se-Se bond, and the -SeH intermediates are more nucleophilic than thiols (-SH) [40,41]. Furthermore, H_2_Se is another crucial metabolite which is involved in the biological properties of organoselenium compounds. H_2_Se is a critical intermediate in Se metabolism, enabling the formation of selenoproteins that protect against oxidative stress [39,42]. Diselenides mainly undergo the following metabolic reactions: reduction, methylation, and incorporation into selenoproteins, among others. The main crucial metabolites involved in the biological activity of selenocompounds are -SeH, which can be further reduced to hydrogen selenide (H_2_Se) and methylselenol (CH_3_SeH) [39,43].

If we focused on ditelluride derivatives, there are few reported compounds because Te derivatives have not been as well-studied as other chalcogens. However, diphenyl ditelluride (DPDTe) has aroused interest among the scientific community because of its effects [44]. Furthermore, its toxicity is associated with its capacity to react with -SH groups from biologically significant molecules as GSH or cysteine [45].

It should be highlighted that the dichalcogenide bond has not only been incorporated into small molecules, but in the past 20 years, dichalcogenide-containing nanomaterials have been investigated as novel materials that can function as nanocarriers for drug delivery due to their unique chemical and biological properties [46]. For example, in the case of nanocarriers, they incorporate Se-Se or Te-Te as a linker or bridge. Chemotherapy has few drawbacks such as poor targeting, side effects, or the production of drug resistance. In this context, nanomedicine has driven the further development of cancer treatment. Nanocarriers based on nanomaterials can improve the targeting of drugs by delivering the drug at the tumor site, increasing their stability, solubility, biodistribution, and pharmacokinetics, reducing the toxicity and achieving higher efficacy with minimal side effects [47]. The diselenide bond has been recently used to design ROS-responsive nanodrug systems due to the fact that this bond can be cleaved by ROS [48]. Interestingly, this bond can also be cleaved by GSH [49]. If the disulfide bond is compared with the diselenide one, the latter bond has a lower bond energy (240 kJ/mol vs. 172 kJ/mol) due to the larger size of Se [50]. Therefore, prodrugs containing diselenide bonds are more sensitive than disulfide-containing ones under different stimuli. The Se-Se bond can be easily cleaved, either by oxidation to form -SeOOH or reduction to form -SeOH in different redox conditions; so, it has been incorporated into polymer-based “on-demand” drug delivery systems [50]. Diselenide bonds respond better to elevated levels of GSH and hydrogen peroxide (H_2_O_2_) in tumor cells, making them more effective for controlled drug release. Furthermore, they have a greater efficacy in tumors with low GSH levels. Unlike disulfide bonds, which may not be effective in tumor cells with low GSH levels, diselenide bonds can be degraded by both GSH and H_2_O_2_, making them more versatile due to their stimuli responsiveness [51,52]. Moreover, diselenide bonds have shown better biocompatibility [53]. Thus, their use as stimuli-responsible carriers is increasing for biomedical applications. Furthermore, ditelluride-containing nanoparticles have recently emerged as an innovative approach for drug delivery systems. The ditelluride bond energy (149 kJ/mol) is lower than the disulfide and diselenide bond due to its bigger radius and weaker electronegativity. Thus, ditelluride-containing polymers are more sensitive to be cleaved [54]. For this reason, it is recently becoming interesting to introduce it as part of nanoparticles for the controlled release of drugs.

In this regard, the main purpose of this review is to collect the most recent studies conducted in recent years on bioactive molecules that incorporate the diselenide or ditelluride bond in their structure. The disulfide bond is excluded from this review since it has been widely studied. Thus, this review includes bioactive diselenides and ditellurides in the form of small molecules and in the form of more complex materials such as nanoparticles.

## 2. Diselenide Derivatives with Biological Activity

Diselenides are a class of Se-containing compounds with a Se-Se bond in their structure. Among the different subclasses of Se-containing compounds, diselenides have been demonstrated to exhibit different biological activities and are promising compounds for treating a plethora of diseases including cancer, bacteria, parasites, viruses, or Alzheimer’s disease (AD).

The use of diselenide bonds for the development of new treatments can be achieved using single molecules and complex materials. This section summarizes the active diselenide compounds that have been published during recent years, sorted by pathology and classified from the simplest small molecules to complex materials such as nanoparticles, nanogels, or micelles.

### 2.1. Diselenides with Anticancer Activity

The interest in Se compounds lies in the variety of biological activities they present. Amidst organoselenium compounds, diselenides have gained significant interest in the field of medicinal chemistry. Among them, a large part of the studies of these compounds are devoted to cancer because of their known anticancer activity. Diselenide compounds achieve cyto-selectivity through various mechanisms. On the one hand, they possess antioxidant and prooxidant activities. They can act as antioxidants by mimicking GPx activity, reducing oxidative stress through the elimination of free radicals. On the other hand, they can also exhibit prooxidant activities at higher concentrations, generating ROS that induce cytotoxicity selectively in cancer cells [55,56]. Furthermore, some diselenides can influence the activity of a wide range of kinases, which is crucial for cell signaling and proliferation. By inhibiting specific kinases, diselenides can selectively target cancer cells that depend on these pathways for growth and survival activity, which is crucial for cell signaling and proliferation [57]. Diselenides can also trigger apoptosis in cancer cells through various pathways, including the activation of caspases, modulation of mitochondrial permeability, and regulation of apoptotic genes [58]. Certain diselenides can also target angiogenesis, thus selectively affecting cancer cells [59]. The Se-Se bond can be found in small molecules, although, due to its great potential and its properties, it has recently also been included as part of more complex structures such as nanoparticles.

#### 2.1.1. Small Molecules

**Figure 1:**
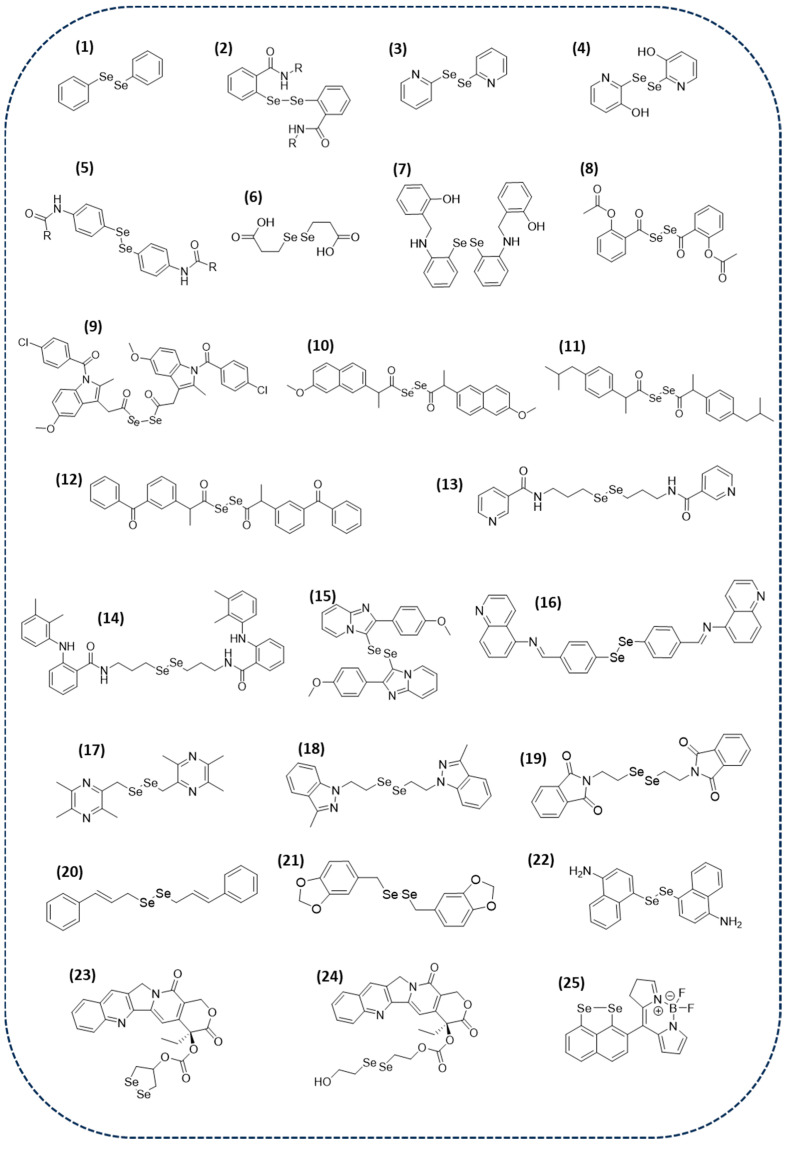
Chemical structure of small diselenide-containing molecules with anticancer activity.

Diphenyl diselenide (DPDSe) (**1** in Figure 1) is a simple and stable organoselenium compound. It is commonly used as a synthetical electrophilic reagent, but its biological activity is of great interest. In the last decade, several studies revealed that it is a good candidate for therapeutic purposes, specifically in cancer research [60,61,62,63]. Recently, DPDSe has been studied against triple-negative breast cancer (TNBC). This compound caused a statistically significant decrease in the viability of MDA-MB-231, BT-549, and MCF-10A cell lines, with IC_50_ values of 60.79, 50.52, and 56.86 μM, respectively. Furthermore, it also caused a decrease in the percentage of colonies formed, demonstrating its potential against breast cancer [64]. Owing to all the evidence regarding the anticancer activity of DPDSe, several studies were conducted on the synthesis and biological characteristics of DPDSe derivatives. Particularly, N-substituted benzisodiselenazol-3(2H) derivatives (**2** in Figure 1) were synthetized and tested as antioxidant and anticancer agents. The results from this study revealed that the anticancer activity of these derivatives was selective towards HL-60 cell lines [65]. Another DPDSe derivative, 2,2′-dipyridyl diselenide (**3** in Figure 1), has been shown to be cytotoxic in human non-small-cell lung carcinoma (A549), with an IC_50_ value of 8.5 μM. However, it does not exhibit a selective toxicity toward cancer cells. Some studies have been performed in order to understand the mechanism of 2,2′-dipyridyl diselenide. The results revealed that it produces intermediates that induce ROS scavenging, the inhibition of redox enzymes, and reductive stress, resulting in a reduction in the GSSG/GSH ratio. Consequently, this cascade activates ER stress-mediated apoptosis, causes DNA damage, and leads to G1 cell cycle arrest [66]. Given the potential of the 2,2′-dipyridyl diselenide scaffold, a new derivative, 2,2′-diselenobis(3-pyridinol) (**4** in Figure 1), was synthesized, and its cytotoxic effect was studied. However, the new derivative did not improve toxicity or selectivity compared to 2,2′-dipyridyl diselenide [67].

Since Se derivatives have been demonstrated to be potent antitumor agents, other different diselenide-containing compounds have been recently studied. Some authors have reported a library of amide and phosphoramidate derivatives which presented a high cytotoxic effect toward several breast cancer cell lines; among them, compound **5** (Figure 1) is highlighted. This compound has shown GI_50_ values lower than 1 μM, meaning that it is three times more potent than cisplatin, making it a promising diselenide candidate for further development [68].

The derivative 3,3′-diselenodipropionic acid (DSePA) (**6** in Figure 1), a structural analogue of selenocysteine, is a well-known diselenide with multiple pharmacological activities. It has been widely studied for its antioxidant, radioprotective, nitric oxide generating, and neuroprotective activities. Previous studies have reported its LD_50_, biodistribution, and pharmacokinetics [69,70]. All this available data prompted its study as an anticancer agent. Particularly, its effect against lung cancer cells has been recently studied. DSePA has been demonstrated to be a concentration and time-dependent cytotoxic compound in human lung cancer cell line A549. Its cytotoxic effect was preceded by a reduction in the basal ROS level and a simultaneous increase in cellular levels of GSH/GSSG and NADH/NAD. These studies finally suggest that DSePA triggers apoptosis through a p53-independent pathway, which makes it a promising candidate for further treatments for lung cancer [71].

An aminophenolic diselenide (**7** in Figure 1) has been proposed as a novel compound for cancer treatment. The design strategy of this compound is based on the generation of ROS from oxygen gas by the synthesized aminophenolic diselenide. There is mounting evidence that the utilization of ROS produced within cancer cells from molecular oxygen is a novel strategy for cancer treatment and has an enhanced cytotoxicity toward cancer cells. ROS detection assays revealed that those novel compounds generate ROS. Nonetheless, aminophenolic diselenides substituted with electron-donating groups in the aniline ring favor aerial oxidation. Since the synthetized organo-diselenides generate ROS, they were screened against HeLa cancer cells, showing a selective cytotoxicity. Furthermore, they can also be used in combination with chemotherapeutic drugs. In view of the results, these diselenide derivatives would be promising candidates for further developments [72].

Other scaffolds that have attracted great attention in cancer research are nonsteroidal anti-inflammatory drugs (NSAIDs). Several studies have documented that a correlation exists between the exposure to NSAIDs and reduced cancer recurrence [73,74,75]. Recently, the first library of a few NSAID derivatives containing Se in the form of diacyl diselenide has been reported (**8**–**12** in Figure 1). Their antiproliferative activity was evaluated in different cancer cell lines, demonstrating that they are more potent than their NSAID counterparts. Compound **11** was not only the most potent antiproliferative compound, with an IC_50_ value below 10 μM in all the tested cancer cell lines, but also exhibited selectivity towards cancer cells. These findings illustrate that the approach of incorporating both NSAIDs and diselenide moieties in the design of novel compounds might yield biologically active agents within a feasible framework [76].

An additional study was conducted in order to compare the anticancer effects of selenocyanates and diselenides, both classes of compounds being synthesized and evaluated against several human cancer cell lines. Although selenocyanates exhibited higher anticancer activity than their diselenides analogues, two of the proposed diselenides (**13** and **14** in Figure 1) had IC_50_ values below 20 μM in all the studied cancer cell lines (Caco-2, BGC-823, MCF-7, and PC-3) [77].

Amidst organoselenium compounds, heteroaryl selenides and diselenides have drawn significant interest in medicinal chemistry due to their diverse range of biological activities. For example, some diselenides containing pyridyl rings have been reported to inhibit acetylcholinesterase (AChE) [78], whereas quinoline derivatives exhibited antioxidant activity [79]. Inspired by the potential of this class of compounds, few groups have recently reported novel bioactive derivatives containing an heteroaryl diselenide scaffold in their structure. Particularly, bis(2-arylimidazo[1,2-a]pyridin-3-yl) diselenides have been synthesized and their anticancer activity has been evaluated. Among all the compounds, derivative **15** (Figure 1) demonstrated remarkable anticancer efficacy against a range of cancer cell types, with minimal toxicity towards non-cancerous cells. These findings suggest that diselenide **15** holds promise as a potential therapeutic compound for cancer treatment [80]. On the other hand, a bis(p-formylphenyl)diselenide-based quinoline (**16** in Figure 1) probe has been recently synthesized and studied for the selective and sensitive detection of superoxide species via fluorescence. The idea of merging a diselenide fragment and quinoline in the same chemical structure was inspired by the known anticancer effects of both moieties. Moreover, these compounds have been demonstrated to have stronger anticancer activity than the standard drug cisplatin [81].

A novel ligustrazine diselenide derivative, 1,2-bis ((3,5,6-trimethylpyrazin-2-yl) methyl) diselenide (**17** in Figure 1), has been recently synthesized and studied in vitro in A549 lung cancer cells. This compound has remarkable cytotoxic effects on A549 cancer cells, with an IC_50_ value of 6.8 μM. Furthermore, this study also revealed that this compound induces apoptosis and inhibits the proliferation, migration, invasion, and colony formation of those cells [82].

It is well-known that allosteric glutaminase inhibitors are optimal frameworks to be further developed as glutamine-dependent anticancer agents. However, their clinical application is still not very clear, given that their in vivo efficacy is quite poor in the sense that they show partial inhibition. In order to develop potent irreversible kidney type glutaminase (KGA) allosteric inhibitors, a diselenide motif was incorporated in the middle chain of those allosteric inhibitors. Among the tested diselenide compounds, two of them (compounds **18** and **19** in Figure 1) have showed a potent inhibition of KGA (IC_50_ values of 0.2 and 0.4 μM, respectively). Diselenide compounds were also tested against A549 and H22 lung and hepatocellular carcinoma cancer cell lines, with compound **19** showing IC_50_ values of 0.83 μM and 1.6 μM, respectively. Studies of the mechanism of action of these compounds revealed that the diselenides interact with Lys320 residues of KGA. These results demonstrate that the inclusion of diselenide to KGA allosteric inhibitors could significantly improve their in vivo anticancer efficacy [83].

Among the recent synthesized diselenide compounds, two derivatives, cinnamyl and a benzodioxyl analog, stand out as anticancer agents (**20** and **21** in Figure 1) [84]. The incorporation of a Se-Se bond to those scaffolds resulted in compounds **20** and **21**, which were evaluated in colon (HT-29, HCT-116), lung (H1299, HTB-54), and breast (MDA-MB-231, MCF-7) cancer cell lines. The results showed that these compounds were active in all the tested cancer cell lines except for MCF-7. It is worth mentioning that compound **21** was not only the most active compound in three of the tested cell lines (IC_50_ values of 10.1, 11.3, and 3.6 μM in HT-29, HCT-116, and H1299 cell lines, respectively), but also was not toxic for non-malignant cells.

Interestingly, novel naphthalene-based diselenides have been reported to exhibit in vitro anticancer activity. Among these novel compounds, derivative **22** (Figure 1) demonstrated a pronounced anticancer activity against breast cancer cells (IC_50_ = 11.8 µM), with minimal cytotoxicity to the primary fibroblasts (no growth inhibition was observed) [85].

The formation of seleno-prodrugs is a valid strategy for improving the activity of drugs. Particularly, two diselenide prodrugs of camptothecin (**23** and **24** in Figure 1), a topoisomerase I inhibitor, have been recently reported. The cytotoxicity of these prodrugs was significantly higher compared to the parent drug. The prodrug activation process led to the generation of -SeOH intermediates, facilitating the continuous conversion of GSH and oxygen into oxidized glutathione (GSSG) and superoxides. The data obtained from this study suggest that the integration a Se-Se bond into a drug may increase the drug’s efficacy [86].

As it has been mentioned above, a plethora of research has been dedicated during recent years towards diselenides as anticancer agents. However, these derivatives have not only received enhanced attention as anticancer agents, but also, they have been studied in medicine as molecules for monitoring cancer. Specifically, the first cyclic diselenide probe for the selective detection of superoxides has been recently developed [87]. The generation of ROS is linked to various human disorders, in which cancer is included. Hence, the selective and sensitive detection of those ROS with fluorescence microscopy is a strategy that can be carried out with organic molecules. A new diselenide probe consists of a cyclic diselenide containing BODIPY (4,4-difluoro-4-bora-3a,4a-diaza-s-indacene) (**25** in Figure 1). This probe has demonstrated a selective response of superoxides over other ROSs. Furthermore, results in breast cancer cells suggest that this probe could be used in vivo for the detection of superoxides.

#### 2.1.2. Metal Complexes

In recent years, a wide variety of studies have supported the use of metal-based complexes as potential drugs with different bioactivities such as anticancer [88,89], antiparasitic [90,91], or anti-neurodegenerative activities [92,93], among others. Within the different metal elements involved in the formation of metal complexes with bioactivity, a wide variety of Se-containing ligands have proven to be a valid strategy for developing therapeutic drugs [94].

**Figure 2:**
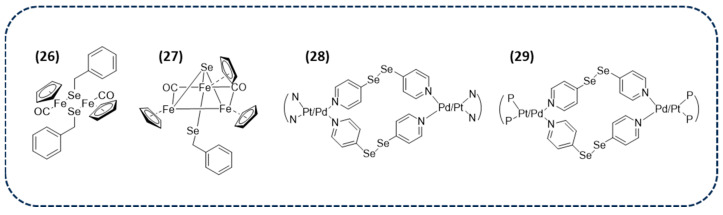
Chemical structure of diselenide-containing metal complexes with anticancer activity.

Among them, cyclopentadienyl diiron carbonyl complexes containing selenocompounds as ligands, have recently been synthetized and biologically evaluated for that purpose (**26** and **27** in Figure 2). As can be seen in their structure, these complexes have been proposed as carbon monoxide (CO)-releasing molecules that can be used for cancer treatment. The cytotoxicity of both compounds was evaluated via the MTT assay in human breast and colon cancer cell lines, showing that both compounds were cytotoxic with an increased cytotoxic activity with UV radiation in some cases [95].

Interestingly, transition metal-based macrocycles are known to have applications in homogenous catalysis [96], although they are emerging as potential anticancer agents. Their antiproliferative profiles have been ascribed to the structural and DNA-binding properties [97,98]. This fact has been linked to the known anticancer property of Se, and has led to the synthesis and biological evaluation of palladium and platinum macrocycles of 4,4′-dipyridyldiselenides, particularly with ethylenediamine and tetramethylenediamine capping ligands (**28** in Figure 2). These stable and water-soluble macrocycles exhibit an anticancer effect in A549 (lung) and MCF7 (breast) cancer cell lines, where palladium complexes demonstrate favorable activities (IC_50_ = 1.7–19 µM) compared to the reference compound cisplatin (IC_50_ = 22 µM and 32 µM in MCF7and A549 cancer cell lines, respectively) [98].

In addition to 4,4′-dipyridyldiselenide metallo-macrocyclic complexes of palladium and platinum, other derivatives were studied owing to the previously reported anticancer potential. Specifically, complexes with diphosphines and xantphos (**29** in Figure 2) were investigated because of their ability to produce stable complexes. Biological experiments were conducted in human breast, lung, bone, and ovarian carcinoma cells. The IC_50_ values were close to those of the refence compound cisplatin. On the other hand, they also induced a greater number of nuclear abnormalities than cisplatin. Thereby, the higher activity of macrocycles, which could be due to their ability to retain cancer cells compared to smaller molecules, may open a new pathway for exploring metal-based anticancer agents [99].

#### 2.1.3. Complex Materials

The compounds reported in the previous sections incorporate the diselenide bond as part of the core of the organic molecule or as part of the coordination complex. However, due to new advances in cancer research, recent studies have incorporated the diselenide moiety in more complex materials such as nanoparticles, nanogels, polypeptides, micelles, or linkers.

Nanomaterials have been studied for many years for drug delivery. However, their primary function is to act as carriers, ensuring the efficient and targeted transportation of drugs. For oncology applications, these nanomaterials must not only serve as carriers, but also, they should possess a certain anticancer capacity, such as increasing hypoxia in the tumor microenvironment. In view of the former, diselenide-containing drug delivery systems have emerged as promising candidates for this purpose. The Se-Se bonds of these systems can be easily oxidized. As a result, diselenide-containing nanomaterials have a dual function: they serve as delivery platforms and as enhancers of anticancer properties [100]. Previously, the nanoparticles reported for anticancer treatments were based on disulfide bonds. However, the diselenide bond has emerged as a better alternative. The lower bond energy of diselenide bonds makes them easier to be cleaved, on account of the larger radius and weaker electronegativity of the Se atom [101,102,103]. Furthermore, it is well-known that the breakage of diselenide bonds produces -SeOH, a selenometabolite with well-known anticancer activity [43], when the bond is cleaved by both GSH and ROS in the cytosol, although some studies suggest that this diselenide bond is primarily cleaved by GSH, not ROS [51].

In this section, the advances in recent years of these types of complex materials are compiled.

#### Nanogels

**Figure 3:**
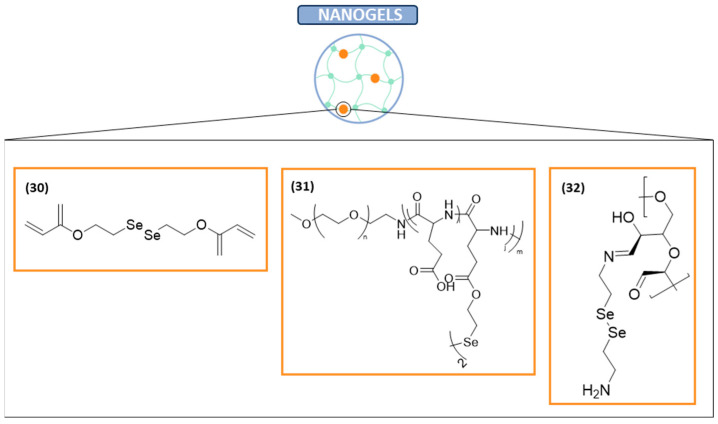
Diselenide-containing nanogels with anticancer activity.

Nanogels are a variety of nanocarriers designed to enhance drug delivery, minimize side effects, and improve tumor chemotherapy [104]. Nanogels are three-dimensional networks formed by the physical or chemical cross-linking of hydrophilic or amphiphilic polymer chains [105]. Recently, a diselenide-cross-linked poly(N-vinylcaprolactam) nanogel co-loaded with gold nanoparticles and methotrexate (**30** in Figure 3) was reported as a multifunctional nanoplatform to improve chemotherapy and the imaging of tumors. It has been designed to avoid the toxic effects of chemotherapy and the lack of specificity. This drug delivery system contains diselenide bonds, as they are highly responsive H_2_O_2_ in the tumor microenvironment. This diselenide nanoplatform was successfully developed, demonstrating good colloidal stability and adequately releasing gold nanoparticles and methotrexate for the computed tomography imaging of tumors. Furthermore, this nanoplatform has demonstrated to improve tumor chemotherapy through the regulation of the tumor microenvironment via tumor-associated macrophage remodeling, as demonstrated in vivo, where tumor growth inhibition was achieved (relative tumor volume = 5.3). Therefore, this diselenide-containing nanoplatform could be a promising nanomedicine formulation for tumor chemotherapy [106].

In the context of the ongoing strategy of using nanogels to enhance the potential of chemotherapy and radiotherapy and reduce the side effects, a recent study has developed a polypeptide nanogel containing an X-ray responsive diselenide bond (**31** in Figure 3) for the on-demand delivery of chemotherapeutic agents. Doxorubicin (DOX) was encapsulated into the core of the polypeptide nanogel, which was released due to the diselenide bond degradation. This nanogel, which was studied in human A549 lung carcinoma-bearing nude mice, provides a significant synergistic antitumor effect as a result of the combination of both chemotherapy and radiotherapy. Hence, it could have a potential application in clinical settings [107].

In the search for new strategies for cancer treatment, GSH consumption-enhanced cancer therapies have emerged as a significant approach to decompose glucose into gluconic acid and H_2_O_2_ to starve tumors. A recent study has reported a diselenide-functionalized dextran-based hydrogel (**32** in Figure 3). This hydrogel is able to degrade and rapidly release the loaded drugs by increasing acidity and H_2_O_2_ during glucose oxidase-mediated tumor starvation and hypoxia-activated chemotherapy. As a result of the overproduced H_2_O_2_, accelerated intracellular GSH consumption is caused due to the catalytic effect of the released diselenides from the degraded hydrogel. Hence, there is an enhanced anticancer effect [108].

#### Nanophotosensitizers

Nanotechnology has also been applied to phototherapy, particularly to photodynamic therapy, which consists in the irradiation of abnormal cells using photosensitizers and light sources. In phototherapy, light is used to kill the cancer cells using a photosensitizer, which is a drug that produces cytotoxic species after absorbing light photons [109,110].

**Figure 4:**
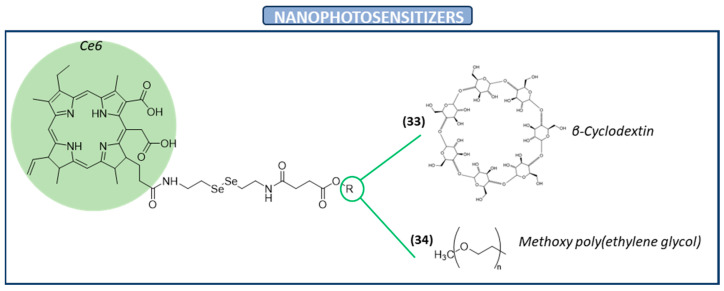
Diselenide-containing nanophotosensitizers with anticancer activity.

In the absence of light, photosensitizers have a minimal impact on the viability of normal cells since they become active only upon exposure to light, generating ROS and selectively eliminating cancer cells. However, they are normally disseminated through the whole body due to their low specificity against cancer cells. Therefore, novel vehicles are needed to solve this drawback [111]. A recent study has reported chlorin e6 (Ce6) as a nanophotosensitizer against cervical carcinoma cells. In this study, Ce6 was conjugated with succinyl β-cyclodextrin via diselenide linkages (**33** in Figure 4). The objective of using a diselenide linkage is based on its ability to be cleaved by ROS in cancer cells. The in vitro results revealed that the cytotoxicity of the nanophotosensitizer was significantly higher than those of Ce6 itself. Furthermore, it was also tested in vivo and the nanophotosensitizer showed a more potent inhibition efficacy than the Ce6 alone. Moreover, this nanophotosensitizer showed a specific delivery capacity and phototoxicity. Due to these facts, this novel nanophotosensitizer of Ce6 is a promising candidate for cervical cancer treatment [112].

A new nanophotosensitizer containing Ce6 and diselenide linkages has been developed. Specifically, this nanophotosensitizer was composed of methoxy poly(ethyleneglycol) (mPEG), Ce6, and phenylboronic acid pinacol ester with diselenide linkages (**34** in Figure 4). This nanophotosensitizer was designed for the purpose of being used in a ROS-sensitive photodynamic therapy for cervical cancer. The results showed that, in effect, it supported the ROS-sensitive delivery of Ce6 against cancer cells. Moreover, in vivo tumor models of HeLa cells revealed that this nanophotosensitizer had sensitivity against oxidative stress in tumor tissues. Once again, this suggests that nanophotosensitizers containing diselenide linkages are promising vehicles for cervical cancer treatment [113].

#### Mesoporous Organosilica Nanoparticle

**Figure 5:**
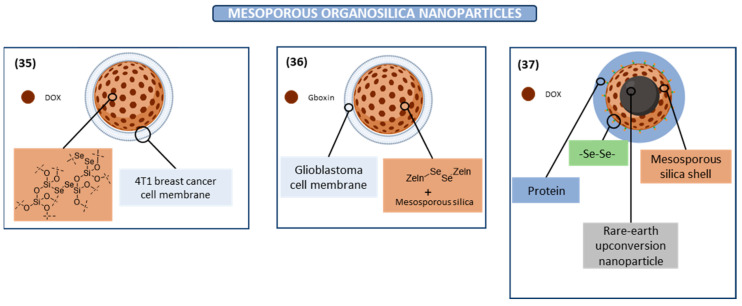
Mesoporous organosilica nanoparticles (MONs) with anticancer activity.

Mesoporous organosilica nanoparticles (MONs) have recently emerged as attractive materials for drug delivery due to their characteristics such as large surface area, biocompatibility, tunable structure, and controllable degradation [114]. This controllable drug release from the MON can be achieved by different degradation pathways, and the X-ray radiation response has been highlighted. Taking this into account, a biomimetic diselenide-bridged MON (**35** in Figure 5) has been recently proposed, since the diselenide bond is sensitive to X-ray radiation. Specifically, this drug delivery system has been studied in the case of DOX and PD-L1 immune checkpoint blockades. n vivo experiments showed a greater accumulation of these DOX-loaded nanoparticles at the tumor site and a prolonged blood circulation time due to diselenide bond cleavage with a low dose of X-ray radiation, which enhances immunogenic cell death at the tumor site [102].

Glioblastoma is the most prevailing primary brain tumor, presenting poor therapeutic prognosis due to factors such as the low rate of drugs crossing the blood–brain tumor barrier, low selectivity, and high toxicity of chemotherapeutic drugs. Interestingly, MONs have also been recently studied in glioblastoma for the purpose of finding an appropriate treatment capable of solving all these impediments. CUDZG, a novel dual-functional nanoparticle for imaging and glioblastoma therapy has been developed. This nanoparticle was formed by Gboxin, a newly developed drug for glioblastoma, and a ZnGa_2_O_4_:Cr^3+^, Sn^4+^ (ZGOCS)-containing mesoporous silica. Diselenide bonds, particularly diselenide bonds conjugated with zein molecules, are used as bridge in these nanoparticles (**36** in Figure 5). The reason for using these diselenide bonds is related to its ability to be reduced to -SeOH by GSH and oxidized to -SeOOH by ROS. Consequently, these diselenide-containing drug carriers can achieve the release of the drug at the tumor site, improving the efficacy and reducing the toxicity of the drug [115]. In view of the application of diselenide-containing MONs as a nanovehicle for the release of drugs, a recent study has reported its application for the release of DOX drugs (**37** in Figure 5), showing an inhibitory effect on tumor growth and negligible damage to normal tissues. Hence, they have promising prospects in targeted drug delivery for cancer chemotherapy [116].

#### Nano-Assemblies for Combined Chemoradiotherapy

**Figure 6:**
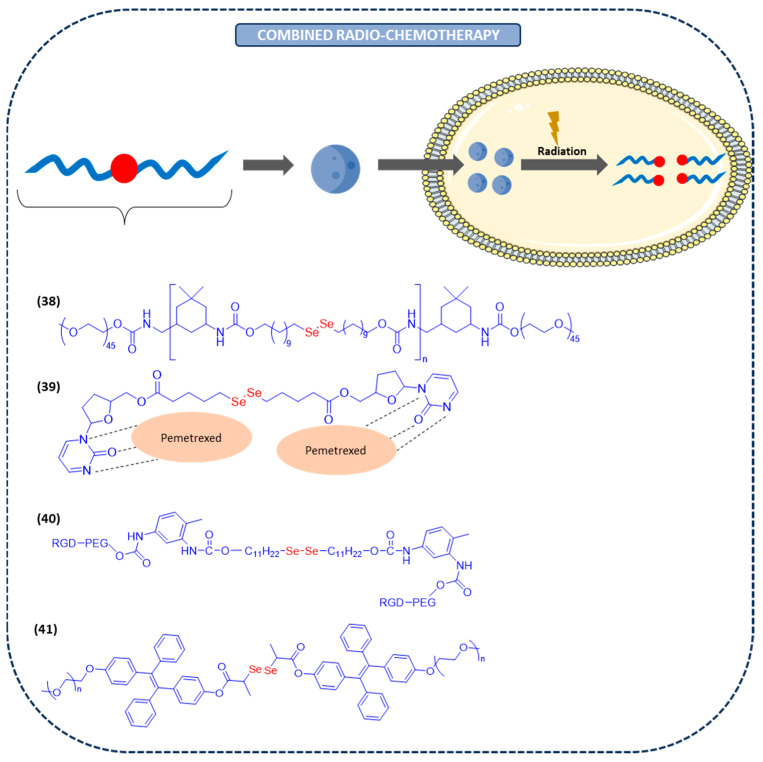
Nanoparticles for combined radio- and chemotherapy.

Among the diverse types of nanocarriers for anticancer drug delivery, X-ray-responsive nanocarriers have shown great promise for the efficacy of chemoradiotherapy. Radio- and chemotherapies are the two most common treatments in oncology. By applying both types of therapies, the antitumor efficiency could be reinforced [117]. However, superimposing both treatments to a patient increases the biotoxicity of both, making this combined treatment unacceptable for some patients [118]. Consequentially, radiation-responsive nanocarriers have arisen as a strategy to load and transport anticancer drugs to the tumor site. Among them, diselenide-based nanocarriers have shown excellent biocompatibility and biodegradability. Furthermore, the released products have shown a synergistic antitumor effect and immunomodulatory activity. Recently, a novel drug delivery system based on a diselenide block copolymer as the nanocarrier has been developed (**38** in Figure 6). Diselenide linkages can undergo gradual oxidation by H_2_O_2_, resulting in the dissociation of the polymer chains and the rupture of the Se nanoparticles. This nanocarrier is not only able to suppress the toxic effects of chemoradiotherapy but also increases the antitumor efficacy [119].

Ongoing with this strategy and on account of the limitations of the antitumor immunotherapies, a diselenide–pemetrexed assembly, which combined immunotherapy, radiotherapy, and chemotherapy in a single system, has been recently developed. The creation of this innovative nano-assembly involves the co-assembly of pemetrexed and cytosine-containing diselenide through hydrogen bonding. These hydrogen bonds can be cleaved under γ-radiation, thus releasing pemetrexed (**39** in Figure 6). Simultaneously, diselenide can be oxidized to seleninic acid, hence suppressing the expression of human leukocyte antigen E in cancer cells and activating the immune response [120]

In the last two decades, the use of natural killer (NK) cell-mediated immunotherapy in combination with radiotherapy and target therapeutics is increasing. In a recent study, diselenide-containing nanoparticles for the delivery of DOX have been developed (**40** in Figure 6). Radiation facilitates the release of the chemotherapeutic drug from the nanoparticle at the tumor site by systemic administration, thereby increasing the efficacy. Furthermore, due to the presence of diselenide bonds in the nanoparticles, it can be oxidized to -SeOOH and induce a synergistic antitumor effect and enhance the immunomodulatory activity by improving the function of NK cells. For this reason, diselenide-containing nanoparticles could be a potential approach to achieve simultaneous treatments of immunotherapy, chemotherapy, and radiotherapy [121].

Interestingly, another recent study has developed Se-containing carrier-free assemblies with aggregation-induced emission properties, which combines cancer radiotherapy with chemotherapy. The γ-radiation is able to cleavage the diselenide bonds, thus releasing all the assembles (**41** in Figure 6). Furthermore, as diselenide bonds are used as linkers, -SeOOH is consequently formed, leading to the combination of radiotherapy and chemotherapy [122]. Regarding γ-radiation, it has been shown that γ-rays generate an aqueous solution of ROS containing species such as ·OH, ·O_2_H, and H_2_O_2_, which break the diselenide bonds of the nanoparticle [39].

#### Micelles

In addition to all the nanocarriers mentioned previously, which have succeeded in enhancing the characteristics of anticancer treatments, other different polymeric materials have arisen as potential therapeutic carriers with biomedical applications. Among them, polymeric micelles are becoming increasingly popular due to their various advantages, including improved aqueous solubility, prolonged drug retention time in plasma, low toxicity, and selective enhancement within tumor areas through the enhanced permeability and retention effect [123].

**Figure 7:**
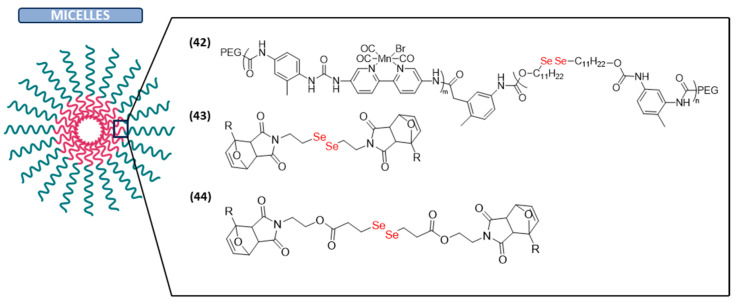
Diselenide-containing micelles with anticancer activity.

Among the various applications for which they have been used in recent years, their application in CO therapy is worth mentioning. CO-based gas therapy can induce the dysfunction of mitochondria via increasing the level of ROS in cancer cells, thus suppressing the tumor growth. However, this therapy has some drawbacks such as poor tumor targeting ability, strong affinity toward hemoglobin, or low effects on solid tumors. Therefore, H_2_O_2_-responsive diselenide-containing micelles to combine CO therapy with chemosensitization and antiangiogenesis therapies have emerged as an integrated solution (**42** in Figure 7) [124]. On the other hand, core-cross-linked micelles have gained a lot of attention as drug delivery systems in contrast to polymeric micelles because they offer several advantages such as improved colloidal stability, biocompatibility, stimuli control, high drug-loading capacity, and prolonged drug release [125]. Particularly, diselenide and disulfide redox-responsive core-cross-linked micelles incorporating doxorubicin, an anticancer drug (**43** in Figure 7), have been recently synthesized and evaluated in vitro against BT-20 cancer cells. The results showed that the developed micelles were non-toxic against HEK-293 normal cells (85% cell survival in the presence of up to 200 μM of micellar dosage). Furthermore, both micelles exhibited potent cytotoxicity against BT-20 cancer cells (IC_50_ = 19.90 and 17.90 μM for disulfide and diselenide micelles, respectively), with the disulfide-containing micelles releasing less DOX than the diselenide ones. The results of this study show the potential of diselenide-containing core-cross-linked micelles as intelligent carriers for anticancer drug delivery vehicles [52].

In a similar way, near-infrared (NIR)-responsive interface cross-linked micelles containing the 3,3′-diselanediyldipropionoate cross-linker were prepared for the release of DOX (**44** in Figure 7). These DOX-loaded micelles were demonstrated to be pH and NIR-responsive. Furthermore, in vitro toxicity studies revealed that they present a higher antitumor activity toward HeLa cells than to non-cancerous cells. Therefore, these micelles could be used as promising drug delivery systems for cancer treatment [126].

#### 2D Nanomaterials

Two-dimensional atomic crystals have recently emerged as new nanoagents for cancer theragnostics due to their large surface area and their strong optical absorbance. Despite their relevant anticancer effect, undesired inflammatory responses commonly appear during hyperthermia due to the intrinsic nanotoxicity of those crystals. Consequently, there is a need to develop novel 2D photonic atomic crystals with intrinsic anti-inflammatory activity. In this context, Nb diselenide nanocrystals have emerged as potential candidates for this purpose due to the known biocompatibility of Nb metal and the anticancer effect of Se. In vitro and in vivo experiments have revealed their low toxicity. Furthermore, subcutaneously implanted tumors were rapidly ablated, and the intrinsic inflammation of the crystal is inhibited through reactive oxygen and nitrogen species scavenging [127].

Various 2D materials, including transition metal dichalcogenides, have been investigated for cancer therapy owing to their ability to be combined with chemotherapeutic drugs and photosensitizing agents.

The research focus on 2D materials is gaining attention due to their properties such as ultrathin structure, photothermal effects, high drug-loading capacity, and biocompatibility. A wide variety of 2D materials, including transition metal dichalcogenides, have been investigated for cancer therapy given their ability to be combined with chemotherapeutic drugs and photosensitizing agents. With this purpose, several nanosheets of poly(ethylene glycol)-block-poly(propylene glycol)-block-poly(ethylene glycol) (PPP) were prepared, particularly PPP-molybdenum disulfide (PPP-MoS_2_), PPP-tungsten disulfide (PPP-WS_2_), PPP-molybdenum diselenide (PPP-MoSe_2_), and PPP-tungsten diselenide (PPP-WSe_2_). These nanosheets were studied in vitro and the results revealed that PPP-MoSe_2_ was biocompatible at 30 µg/mL. Furthermore, these nanosheets exhibited the excellent inhibition of MDA-MB-231 cancer cells at that concentration. Given the potential of this diselenide material, an alginate-chitosan-PPP-MoSe_2_ hydrogel was also developed by this research group, demonstrating its potential for anticancer applications [128].

#### Other Diselenide Nanoparticles

**Figure 8:**
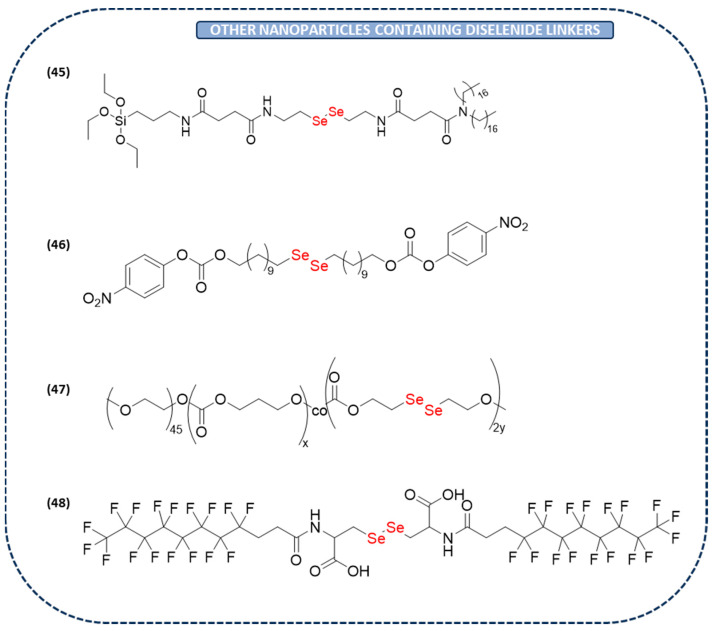
Diselenide-containing linkers in other diselenide nanoparticles with anticancer activity.

The diselenide bond is commonly used as a linker in nanoparticles because it responds to both GSH and ROS. Thus, among its multiple applications, it has recently been used as an intracellular carrier of dehydroascorbate (DHA). In clinical applications, vitamin C has recently surfaced as a promising agent for anticancer purposes. However, the anticancer effect is exerted by dehydroascorbate, the oxidized form of vitamin C. Consequently, given the need to develop strategies for the intracellular delivery of DHA, GSH-sensitive liposomes with a diselenide linkage were used as DHA carriers for cancer treatment (**45** in Figure 8). The results demonstrated that these nanoparticles induced a much higher GSH depletion in HT29 colon cancer cells than the controls, exhibiting significant cytotoxicity against those cancer cells. Hence, liposomes containing diselenide may hold significant promise as carriers for delivering DHA in cancer treatment [129].

For the first time, and continuing with drug delivery systems, a small molecular prodrug of paclitaxel–oxaliplatin was synthesized with covalent diselenide-containing cross-linking, rendering in a specific way a diselenide nanoprodrug (**46** in Figure 8). This nanoprodrug showed high values of tumor-specific accumulation, drug encapsulation, and deep penetration. Due to the presence of the diselenide bond, this nanoprodrug could consume GSH in the TME and initiate the generation of reactive ROS after being exposed to laser irradiation, which ultimately achieve a controlled release of the drugs. Furthermore, in vivo experiments showed that it inhibits tumor growth and prolongs the survival, turning this nanoprodrug into an attractive approach for cancer immunotherapy [130].

One of the principal drawbacks of most anticancer drugs, including paclitaxel, is their hydrophobicity. Therefore, loading the drugs into water-soluble nanocarriers is of great importance. Particularly, a GSH-responsive paclitaxel-loaded polymer dot with a mitochondria-targeting capability has been developed. This novel nanocarrier contains a diselenide linkage cleaved in the presence of H_2_O_2_ and GSH for a controllable release of paclitaxel. Implementing this method could serve as a prospective strategy to augment the therapeutic effectiveness of cancer drugs [131].

All of the already-mentioned examples within this section use nanoparticles for the release of certain drugs with the aim of improving their properties. However, recently, some diselenide-containing nanoparticles have been demonstrated to possess great anticancer potential without incorporating a drug inside. Particularly, an amphiphilic poly(diselenide-carbonate) copolymer has been designed and synthesized to build spherical nanoparticles (**47** in Figure 8). The anticancer activity of this nanoparticle is related to its Se content, as it can selectively induce cancer cells to express excessive ROS, leading to cellular apoptosis. Furthermore, in vivo studies have demonstrated high efficacy and scarce side effects. It was observed that there was a decrease in the relative tumor volume from **18** in the control to **13** after 12 days of treatment [132].

On the other hand, a novel supramolecular fluorinated amphiphilic diselenide nanoparticle has been recently studied for the first time (**48** in Figure 8). This nanoparticle possesses redox-active properties that autocatalytically decompose H_2_O_2_, leading to the formation of seleninic acid. The outcomes revealed that this diselenide-containing nanoparticle presents a selective cytotoxic effect in the MDA-MB-231 cancer cell line. Furthermore, this nanoparticle showed in vivo efficacy in a mouse tumor model. These results suggest that this diselenide system could be a promising approach for targeting tumor growth and preventing tumor recurrence [133].

#### Nanoparticles for Diselenide-Containing Derivatives

**Figure 9:**
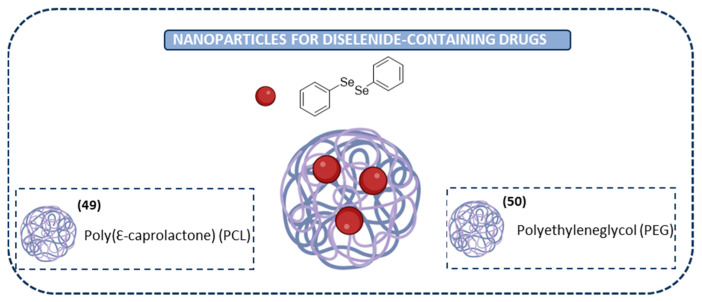
Nanoparticles for diselenide-containing drugs.

The nanoparticles gathered in the previous section are characterized by the presence of the diselenide bond as part of the core of the nanoparticles (as a linker); these nanoparticles have been used in the vast majority of the studies reported in the last few years, as they serve as efficient delivery vehicles with anticancer efficacy. However, there are some reports of the nanoencapsulation of diselenide-containing compounds. This is the case for DPDSe. As it has been highlighted in the previous section, DPDSe is a known organoselenium compound known for its pharmacological properties, including anticancer properties. Numerous studies have demonstrated that DPDSe exhibits effectiveness against various tumor cells. However, it presents an increased toxicity and physicochemical limitations, including poor aqueous solubility, high Log D values, and elevated plasma protein binding rates. In order to solve these drawbacks and to incorporate it to a topical formulation, DPDSe was encapsulated in nanocapsules (**49** in Figure 9). These nanocapsules exhibited higher and more selective cytotoxic effects in malignant melanoma cells than the free compounds (IC_50_ = 47.43 and 65.05 μM, respectively). Furthermore, their physicochemical properties and chemical stability were improved [134].

With the objective of improving the activity and properties of DPDSe, a pegylated formulation was developed in order to achieve an intravenous administration of DPDSe for glioblastoma treatment. Polycaprolactone nanocapsules containing the compound and coated with polyethylene glycol (**50** in Figure 9) were formed and their cytotoxicity was evaluated. The results revealed that the encapsulated compound presented higher thermal stability, and it was hemocompatible. Furthermore, it had a superior in vitro antiglioma effect in comparison to the free compound (IC_50_ = 10 μM and 24.10 μM, respectively). Therefore, the suspension of DPDSe-loaded pegylated nanocapsules can be considered a hemocompatible formulation for intravenous glioma treatment. However, its precise role in crossing the blood–brain barrier must be further investigated [135].

### 2.2. Diselenides with Antibacterial Activity

Selenated compounds have shown a wide variety of biological activities. Given the urgent need to find new antibacterial agents due to the development of drug resistance, several studies have been conducted with selenocompounds as antibacterial agents, with most of them including the following functionalities: selenoesters, selenocyanates, or diselenides [84,136]. Regarding diselenides, not many studies have been reported recently.

**Figure 10:**
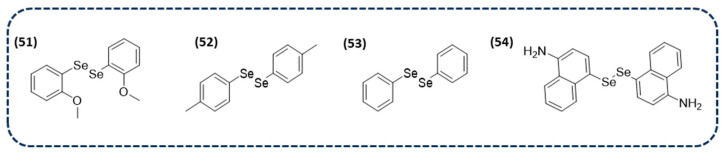
Chemical structures of small diselenide-containing molecules with antibacterial activity.

Among the most recent studies of diselenide-containing compounds with antibacterial activity, some compounds have shown a potent antibacterial activity. This is the case for compounds **51**–**53** (Figure 10). In vitro studies against methicillin-resistant *Staphylococcus aureus* and *Escherichia coli* were conducted. The results revealed that the insertion of o-methoxy and p-methyl groups are able to promote changes in mitochondrial functions, which are associated with antibacterial activity. Mechanistically, these compounds are able to produce damage to -SH groups and promote changes in the mitochondrial functions of bacteria. These results bring a new perspective of the mechanism of action of selenocompounds for the development of novel antibacterial drugs [137].

Furthermore, a novel naphthalene-based diselenide (**54** in Figure 10), which has demonstrated anticancer activity, as indicated in previous sections, has also manifested a potential antibacterial activity against *Escherichia coli* and *Candida albicans*, showing an inhibition zone diameter of 0.83 and 0.82, respectively, compared to the refence drug ampicillin for *Escherichia coli* and clotrimazole for *Candida albicans* [85].

In the search for new alternatives to combat bacterial infections, metabolic homeostasis has arisen as a novel strategy for killing bacteria, especially in implant-associated infections. On the other hand, H_2_Se, a congenic substitute for H_2_S, serves as a bacteria-specific intermediate metabolite, as it is integrated into the H_2_S pathway. Therefore, H_2_Se is able to destroy the bacteria metabolism homeostasis. A nanodrug based on manganese diselenide (MnSe_2_) has been developed to form H_2_Se in bacteria in the acidic microenvironment. This smart nanodrug, which is based on phototherapy, provides a novel strategy to combat bacterial infections [138].

### 2.3. Diselenides with Antiparasitic Activity

There is a growing body of evidence that suggests a connection between Se and antiparasitic activity. Actually, it has been demonstrated that some parasites express selenoproteins and metabolize Se [139]. For these reasons, in recent years, several studies have reported novel selenocompounds as leishmanicidal agents. Among the different Se functionalities, several studies have focused their attention on diselenides, which are presented below.

**Figure 11:**
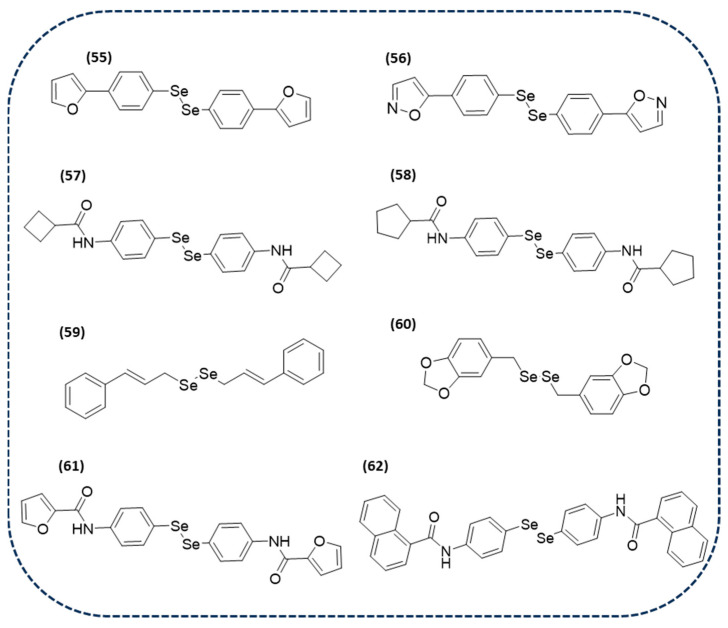
Chemical structures of diselenide-containing molecules with antiparasitic activity.

A recent study has reported several diselenides which have been evaluated in vitro for leishmanicidal activities against *Leishmania infantum* amastigotes. Interestingly, most of the tested derivatives were active in the micromolar range, particularly compounds **55** and **56**, as shown in Figure 11, with IC_50_ values of 0.67 and 0.35 µM, respectively. Additionally, they have been demonstrated to effectively inhibit trypanothione reductase [140].

Several selenocyanates and diselenides were synthesized and screened against promastigotes of *Leishmania major* and *Leishmania infantum*. It is important to highlight that among those compounds, the most potent and less cytotoxic were two diselenides (**57** and **58** in Figure 11), with EC_50_ values of 7.7 and 5.7 µM in of *Leishmania major* amastigotes, and 6.0 and 7.4 µM *Leishmania infantum* amastigotes, respectively. Furthermore, they exhibited higher trypanothione reductase activity than mepacrine. Therefore, these novel diselenides could be promising candidates for treating leishmaniasis [141].

The incorporation of the Se-Se bond in cinnamyl and benzodioxyl has given rise to two new derivatives (**59** and **60** in Figure 11) that not only present anticancer and antibacterial activity, as shown in previous sections, but also present activity against *Leishmania major* and *Leishmania infantum* promastigotes. The obtained results revealed that the diselenide-containing compounds display a moderate activity in both strains, with IC_50_ values of 47.6 and 36.4 µM for compound **59** and 49.4 and 56.1 µM for compound **35**, respectively [84].

Bearing in mind the abovementioned compounds, diselenide-containing small molecules can be considered promising candidates for the treatment of leishmaniasis. Nevertheless, the low solubility and potential instability of these types of derivatives is a major drawback. In an attempt to solve both problems, nanostructured lipid carriers have arisen as a strategy to increase the oral absorption of this type of compound [142]. In a recent study, two diselenides (**61** and **62** in Figure 11) have been encapsulated in glycerol palmitostearate and diethylene glycol monoethyl ether-based nanostructured lipid carriers. The results revealed that the loading of these compounds enhanced intestinal permeability and provided higher plasmatic levels than the effective concentration, which was also corroborated with in vivo studies [143].

### 2.4. Diselenides with Antiviral Activity

In recent years, few studies on diselenides with antiviral activity have been published. Particularly, due to the global SARS-CoV-2 pandemic, a great deal of effort was devoted to the search for new treatments against this viral infection. Furthermore, there is evidence that both selenoproteins and redox-active Se species within the metabolic pool of Se may employ its mechanisms to reduce oxidative stress caused by viruses, mitigate exaggerated inflammatory responses, and address dysfunction in the immune system [144]. For this reason, some diselenides have been mainly studied in this viral infection.

**Figure 12:**
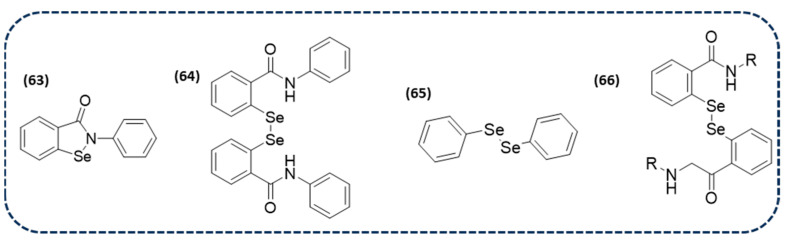
Chemical structure of diselenide-containing molecules with antiviral activity.

It is well-known that the cysteine proteases’ main protease (Mpro) and papain-like protease (PLpro) are important targets for antiviral drugs. Therefore, in silico studies have been conducted to analyze the interactions between Mpro and PLpro and the Se-containing drug ebselen (**63** in Figure 12), along with one of its metabolites and DPDSe (**64** and **65** in Figure 12, respectively). The molecular docking data demonstrated that there could be an interaction between the -SH group of cysteine and the electrophilic region of selenocompounds, leading to a Se-S bond formation, thus inhibiting the enzyme. Furthermore, density functional theory suggests that the -SH nucleophilic attack is more favorable in Se than in another electrophilic group. Therefore, this suggests that some selenocompounds could be further explored as inhibitors of the SARS-CoV-2 protease [145].

Due to the known effect of ebselen as a potent covalent inhibitor of Mpro and PLpro, another study was focused on finding new derivatives for SARS-CoV-2. Particularly, another 34 derivatives based on ebselen, including diselenides (**66** in Figure 12), were screened. The obtained data revealed that, once again, these derivatives are promising candidates for the development of new antivirals targeting the SARS-CoV-2 virus [146].

A further study investigated the mechanisms of SARS-CoV-2 inhibition by DPDSe (**65** in Figure 12), a known therapeutic agent. Firstly, this compound was tested in vitro on SARS-CoV-2 replication, giving CC_50_ and EC_50_ values of 24.61 and 2.39 µM, respectively. These data suggest that it is a good inhibitor of the SARS-CoV-2 virus replication in a cell culture model. Furthermore, in silico molecular dynamics indicated that the adducts between Mpro, PLpro, and DPDSe are stable, with π interactions established between the phenyl ring of DPDSe and residues of histidine and methionine. However, from the mechanistic point of view, the formation of the covalent Se-S bond is energetically less favored than that previously mentioned for ebselen. Nevertheless, further investigations are needed to design novel diselenides as inhibitors of viral proteases for combating SARS-CoV-2 [147].

In addition to SARS-CoV-2, a study has also recently been published on DPDSe (**65** in Figure 12) against the treatment of another viral disease. DPDSe, together with three anti-herpetic drugs, have been evaluated against bovine alpha herpesvirus 2 (BoHV-2), the agent of bovine herpetic mamillitis. DPDSe and cidofovir have been demonstrated to present an antiviral activity against BoHV-2 in vitro. Furthermore, the combination of both compounds has showed a beneficial effect in the progression of BoHV-2 in a sheep model. These results are promising for the use of DPDSe alone or in combination for the treatment of this viral infection [148].

### 2.5. Diselenides for Neurodegenerative Diseases

AD is one of the most prevalent types of dementia. It is an incurable neurodegenerative disease that affects millions of elderly people worldwide. The existing clinical drugs can only slightly relieve the symptoms of AD but cannot cure it. As a consequence, significant efforts have been focused on creating new molecules that target key features associated with a decrease in acetylcholine levels, an increase in oxidative stress, and depositions of amyloid-β (Aβ) and tau proteins [149]. In this regard, Se has emerged as a potential element for the development of new treatments for AD, as they are known for their antioxidant properties and as AChEinhibitors [150,151].

**Figure 13:**
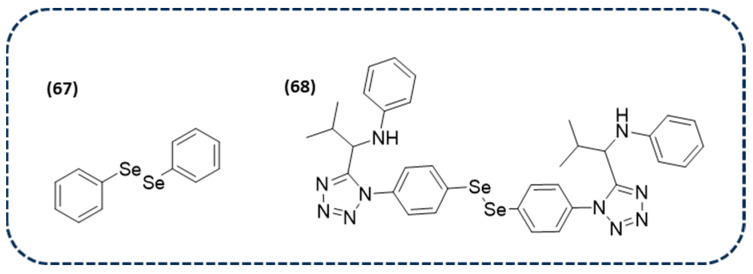
Chemical structures of diselenide-containing molecules for neurodegenerative diseases.

Experimental and clinical studies have demonstrated that the well-known DPDSe (**67** in Figure 13) plays a positive role in cognitive performance through the cholinergic system. This outcome, together with the results obtained in other studies on the ability to inhibit AChE activity in animal models, have led to the exploration of the DPDSe-induced inhibition of AChE for the identification of novel allosteric sites. In silico studies have revealed that DPDSe can strongly bind around the peripheral anionic site, leading to a non-competitive inhibition. These simulations provide mechanistic insights into the type of inhibition of AChE, which can be useful for the development of novel diselenide derivatives [152].

Interestingly, a recent study has reported several compounds as potential AChE inhibitors. From a total of 34 novel synthesized selenocompounds, a tetrazole-based diselenide (**68** in Figure 13) was among the compounds with the highest inhibition efficiency. Furthermore, docking studies showed that this compound is positioned at the catalytic site and the peripheral anionic site, supporting its inhibitory effect [153].

Diselenides have not only arisen as part of small molecules, but a recent study has also incorporated this fragment as a part of a complex nanoplatform. Particularly, an ROS-responsive ruthenium (Ru) nanoplatform drug delivery system for AD management, which promotes neuron regeneration and Aβ clearance, has been designed, synthesized, and studied. The diselenide bond in the nanoclusters can be broken, and the nanocluster can be degraded into small Ru nanoparticles in the Aβ-induced ROS environment. Under NIR irradiation, they can inhibit Aβ formation and disaggregate the preformed fibrils. Furthermore, they can protect and promote neurite outgrowth, and they are able to cross the blood–brain barrier [154].

## 3. Ditelluride Derivatives with Biological Activity

The chemistry of organotellurium compounds has recently evolved, specifically regarding the plausible role of Te in biological reactions and its application to medicine [19]. Several studies have demonstrated that organotellurium compounds present in vitro and in vivo antioxidant enzymatic activity, with better scavenging activity than their organosulfur and organoselenium analogs [45,155]. Nowadays, there is still a minimal understanding of the bioreactivity of Te-containing compounds. However, the study of Te-containing compounds is developing and growing in recent years due to their attractive biomedical applications. Several ditelluride derivatives have emerged, with different bioactivities such as anticancer or antibacterial activities. Furthermore, in addition to studies on small molecules containing ditelluride bonds, several nanomaterials containing Te-Te bonds have been reported. All these novel approaches are summarized in this section.

### 3.1. Bioactive Small Molecules Containing Ditelluride Bond

**Figure 14:**
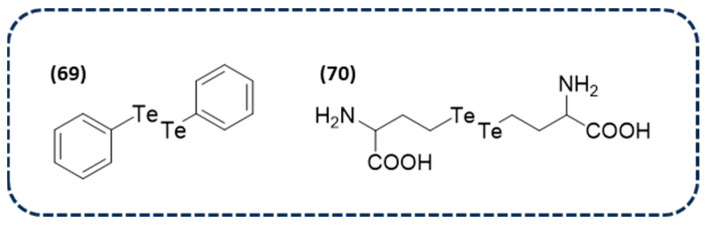
Chemical structures of bioactive ditelluride-containing molecules.

As DPDSe has been widely explored as a diselenide-containing bioactive molecule, its Te analog, DPDTe (**69** in Figure 14), has attracted the attention of some researchers. DPDTe is a small and nucleophilic organotellurium compound. Given the interest that this compound arouses, its toxicological mechanism has been recently studied, not only as an individual compound, but also as a reference to elucidate the cytotoxicity of nucleophilic organometallic compounds. The results revealed that DPDTe exhibited cytotoxicity in vascular endothelial cells and fibroblastic IMR-90 cells. Furthermore, it was demonstrated that the cytotoxicity decreased when Te atoms were substituted by Se or S atoms.

The electronic state of Te atoms in DPDTe plays a significant role in the accumulation and distribution of DPDTe within cells. It is associated with the heterolytic cleavage of the Te-Te bond. The results demonstrated that DPDTe can be more easily reduced than its analog DPDSe. Therefore, both nucleophilic and electrophilic organometallic compounds have been demonstrated to exhibit cytotoxicity by particular mechanisms [156]. Furthermore, the effects of DPDTe have been recently studied against the colon cancer cell line HCT116, showing selectivity towards cancer cells when compared to non-tumorigenic cells. Moreover, it was observed that DPDTe is able to establish covalent complexes with DNA topoisomerase I [157].

On the other hand, DPDTe is able to oxidize sulfhydryl groups from biological molecules. For that reason, it has also been studied as an antibacterial agent against *Escherichia coli*. The findings demonstrated that the exposure to DPDTe induced the formation of reactive species, decreased non-protein -SH levels, and produced changes in transcriptional factor reactions. Thus, DPDTe could be characterized as a multi-target compound which causes a disruption in the cellular oxidative state as well as molecular mechanisms associated with *Escherichia coli* [158]. For those reasons, DPDTe could be a promising multi-target compound for the treatment of several diseases.

Te-containing amino acids have been recently synthesized and studied based on previous evidence on the successful bio-incorporation of Te into proteins and peptides [159,160]. One novel ditelluride-containing amino acid has been recently studied, tellurohomocystine (**70** in Figure 14). This amino acid has demonstrated good in vitro biocompatibility in L929 and NIH/3T3 fibroblast cell lines. Interestingly, it showed anticancer activity on breast cancer cell line MCF-7, with an IC_50_ value of 25.36 µM. Furthermore, this Te-containing amino acid induced apoptosis [161].

### 3.2. Bioactive Complex Materials Containing Ditelluride Bond

A wide variety of clinically used chemotherapeutic agents have limitations, as they are hampered by several factors such as lack of selectivity, reduced anticancer efficacy, or severe toxicity. For these reasons, nanoscale drugs have arisen to overcome these limitations [162]. During recent years, Te-containing nanoparticles have been gaining attention among the dichalcogenide-containing nanoparticles. In comparison with its disulfide and diselenide analogs, the lower electronegativity and longer radius of Te results in a lower bond energy in the Te-Te bond. Therefore, nanoparticles containing ditelluride are more susceptible to undergoing reactions in response to a reducing stimulus [19,54].

**Figure 15:**
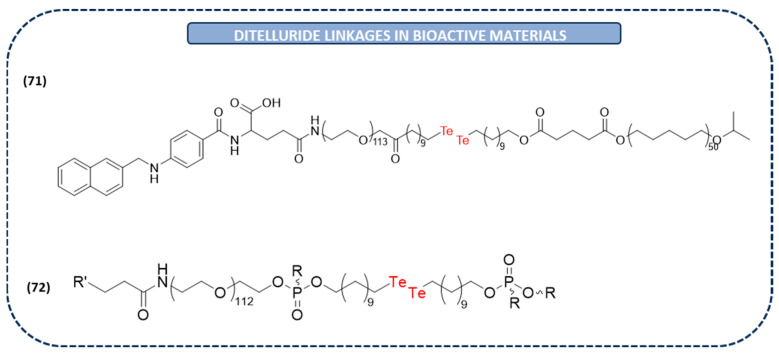
Ditelluride linkages in bioactive nanomaterials.

Thereby, an integrated nanoplatform containing a folic acid ligand and Te-Te bonds has been developed for active tumor targeting and the GSH-responsive drug release of DOX (**71** in Figure 15). The results showed that there was a redox-responsive release of DOX under GSH conditions owing to ditelluride bonds. Furthermore, these nanoparticles led to the drug accumulation and the inhibition of tumor size in vivo on a 4T1 rodent model. These results suggest that this ditelluride-containing nanoplatform could be promising for anticancer drug delivery [163].

As it has been discussed before, X-ray-induced photodynamic therapy is a novel and promising strategy for combined cancer therapy. Consequently, as diselenide bonds, ditelluride bonds have emerged as bridges in these types of nanoparticles for a redox-sensitive release of drugs (**72** in Figure 15). Particularly, these nanoparticles have been designed for the release of verteporfin upon low-dose X-ray irradiation against MDA-MB-231 breast tumors. The GSH-triggered degradation of telluride bridged bonds results in the production of highly cytotoxic radicals that serve as antitumor species, killing the remaining hypoxic tumor cells [164].

## 4. Conclusions and Future Perspectives

Diselenide and ditelluride derivatives have emerged as a novel class of Se- and Te-containing organic molecules to obtain potent therapeutic drugs for multiple pathologies. Based on the latest research, these dichalcogenide bonds are not only considered as part of the structure of small organic molecules, but also, they have been included as part of more complex materials such as nanocarriers for drug delivery or probes for the detection of superoxides. In this review, they have been grouped regarding their structure and biological activity, with the aim of highlighting the importance that the inclusion of this type of link can have in the development of new treatments.

As observed in the extensive bibliography, the importance of the dichalcogenide bond in the development of new treatments for various diseases is demonstrated, particularly in the development of new cancer treatments, which is a major focus of the literature. Among small molecules, the DPDSe compound stands out, as it is one of the most studied diselenides. Furthermore, it has not only demonstrated anticancer activity, as numerous studies show, but it has also been investigated for its effects against other diseases, as well as derivatives of this compound.

Moreover, the Se-Se bond is of great interest in the development of new nanocarriers, as it offers advantages over the disulfide bond due to its lower bond energy. This makes it more sensitive, allowing it to be cleaved by ROS or even GSH in the tumor microenvironment, thereby improving drug efficacy and reducing toxicity to normal cells. As mentioned, the Se-Se bond is present in more complex biomaterials such as nanogels, nanophotosensitizers, organosilica nanoparticles, micelles, and other classes of nanoparticles.

Regarding the ditelluride bond, there is still not much research dedicated to it. However, it has recently attracted more attention due to the obtained results. Although dichalcogenides have demonstrated potential therapeutic efficacy and unique modes of action in preclinical studies, their regulatory status, particularly of diselenides and ditellurides, remains primarily in the research and development phase. Despite encouraging preclinical findings, dichalcogenides have not yet advanced to clinical trials or received regulatory approval for therapeutic use. Ongoing research and clinical trials are necessary to fully elucidate their therapeutic potential and safety profiles before they can be considered for approval by regulatory authorities.

In summary, the information from recent years compiled in this review points out that the dichalcogenide bond could hold the promise to contribute significantly to the development of new treatments targeting various pathologies.
